# Author Correction: Pulsed stimulated Brillouin microscopy enables high-sensitivity mechanical imaging of live and fragile biological specimens

**DOI:** 10.1038/s41592-024-02259-w

**Published:** 2024-04-01

**Authors:** Fan Yang, Carlo Bevilacqua, Sebastian Hambura, Ana Neves, Anusha Gopalan, Koki Watanabe, Matt Govendir, Maria Bernabeu, Jan Ellenberg, Alba Diz-Muñoz, Simone Köhler, Georgia Rapti, Martin Jechlinger, Robert Prevedel

**Affiliations:** 1 Cell Biology and Biophysics Unit, European Molecular Biology Laboratory, Heidelberg, Germany; 2 Collaboration for joint PhD degree between EMBL and Heidelberg University, Faculty of Biosciences, Heidelberg, Germany; 3 European Molecular Biology Laboratory Barcelona, Barcelona, Spain; 4 Developmental Biology Unit, European Molecular Biology Laboratory, Heidelberg, Germany; 5 Interdisciplinary Center of Neurosciences, Heidelberg University, Heidelberg, Germany; 6 MOLIT Institute for Personalized Medicine gGmbH, Heilbronn, Germany; 7 Epigenetics and Neurobiology Unit, European Molecular Biology Laboratory, Rome, Italy; 8 Molecular Medicine Partnership Unit, European Molecular Biology Laboratory, Heidelberg, Germany; 9 German Center for Lung Research (DZL), Heidelberg, Germany; 10 Present Address: Shanghai Institute of Optics and Fine Mechanics, Chinese Academy of Sciences, Shanghai, China; 11 Present Address: Collaboration for joint PhD degree between EMBL and Heidelberg University, Faculty of Biosciences, Heidelberg, Germany; 12 Present Address: Epigenetics and Neurobiology Unit, European Molecular Biology Laboratory, Rome, Italy

**Keywords:** Imaging, Biophysical methods

Correction to: *Nature Methods* 10.1038/s41592-023-02054-z, published online 26 October 2023.

In the version of the article initially published, in Fig. 3c, d and g, the central canal was mislabeled as a blood vessel. In the fourth paragraph of the introduction, first and third paragraphs of the “High-specificity SBS imaging in live zebrafish and *C. elegans*” section and the Fig. 3 legend, mentions of blood vessels have been amended to discuss the spinal chord or central canal regions. Additionally, reference 28 has been changed to Thouvenin, O. et al. Origin and role of the cerebrospinal fluid bidirectional flow in the central canal. *eLife* 10.7554/eLife.47699 (2020). These corrections have been made to the HTML and PDF versions of the article.

