# Supplementary material for: Pulsed stimulated Brillouin microscopy enables high-sensitivity mechanical imaging of live and fragile biological specimens
Source: Nat Methods. 2023 Oct 26;20(12):1971–9. doi: 10.1038/s41592-023-02054-z (PMC10703689; doi:10.1038/s41592-023-02054-z)
Supplement: Supplementary file 1 — Supplementary Notes 1 and 2 and Table 1. [file 41592_2023_2054_MOESM1_ESM.pdf]

nature methods

Article

https://doi.org/10.1038/s41592-023-02054-z

Pulsed stimulated Brillouin microscopy enables high-sensitivity mechanical imaging of live and fragile biological specimens

In the format provided by the authors and unedited

SUPPLEMENTARY INFORMATION

Table of Contents

SUPPLEMENTARY NOTES 2
SUPPLEMENTARY TABLE 4
SUPPLEMENTARY REFERENCES 4

SUPPLEMENTARY NOTES

SI Note 1: Practical limitations to achievable SNR enhancement through quasi-pulsing. Fig. 1 (e) and (f) show that 13 mW average pump power on the sample with 40 ns pulse width and 1.1 MHz repetition rate (duty cycle = 4.4%) has the same SNR as 295 mW CW pump. Therefore, we have demonstrated a pulse enhancement factor of 22.7 (i.e. 1/duty cycle). In our current setup, the narrowest pulse width is ~40 ns, which is limited by the AOMs. Further increases in the enhancement factor can be achieved by reducing the pulse repetition rate. However, this will also reduce the average power (and thus deteriorate the SNR) because of the limited peak pump and probe power. For a 40 ns pulsed setup, the maximum average probe power on the sample is limited to 5 mW because the input power to the fiber-coupled AOM of the probe beam is limited to ~650 mW to prevent damage. The probe AOM has 3 dB insertion loss and a 50:50 fiber coupler is used for coupling some of the probe light out as reference for the balanced detection. The maximum average pump power on the sample is limited to 13 mW for 40 ns pulsed setup. If one further reduces the pulse repetition rate, the average pump and probe power on the sample also decrease proportionally. This would affect the SNR, as the noise is dominated by the input electronic noise of the LIA.

To obtain a high shift and linewidth precision and hence a good Brillouin image, an SNR larger than 30 for water sample is typically needed. For example, as shown in Fig. 1(g), with 20 ms integration time, we use 60 ns pulse so that the probe power on the sample is 7 mW and on the photodiode is 5 mW. Compared to a 40 ns pulsed probe, the increase of the average probe power improves the SNR. The following parameters are used in all our Brillouin imaging experiments for the low power case: 60 ns pulse width, 1.1 MHz repetition rate, 20 mW average pump power and 7 mW average probe power impingent on the sample. This represents an enhancement factor of 15.2 and achieves the same SNR with 300 mW pump and 7 mW probe power for the CW case. It should be mentioned that in order to make a fair power comparison with a total power of ~265 mW[1], we use 700 ns pulse for pump with average power of 243 mW and for probe with average power of 7 mW. This setting is used in this work as 250 mW total power for a high-power case.

In our work, we used AOMs to generate pulses from CW lasers. This results in two limitations. Firstly, the pulse width is limited to several tens of nanoseconds. To generate shorter pulse width such as 1 ns, a fast electro-optic modulator (EOM) could be used. Secondly, the peak power and average power of the pulse is limited by the input power (i.e. the overall laser power). In the future, this could be solved by using an EOM to generate laser pulses at 1560 nm which could then be amplified by an Erbium-doped fiber amplifier (EDFA). The amplified pulse then propagates into a second-harmonic crystal (e.g. a PPLN waveguide) for second-harmonic generation so that a high power, short width 780 nm pulse is generated. A total enhancement factor of 10,000 could be achieved in this way by using 1 ns pulse train with 100 kHz repetition rate for the pump and probe, and 30 kHz (still in the low noise frequency range of the detection system as shown in Extended Data Fig. 2) envelope pump modulation. This would allow it to obtain a similar SNR as the state-of-the-art CW scheme, but for 1 mW pump power and 20 us pixel time only.

SI Note 2: SBS modulation frequency selection and optimal noise bandwidth/scan times. Extended Data Fig. 2 shows the noise spectra of a pulsed probe beam (90 ns pulse width, 1.1 MHz repetition rate) with and without balanced detection (i.e. differential input) measured by a lock-in-amplifier (MFLI, Zurich Instruments). It clearly shows that balanced detection can drastically decrease the noise. For example, when the probe average power on the photodiode

is 12.3 mW, the noise density with balanced detection is 5.7 nV/sqrt(Hz) which is ~10 times smaller than with unbalanced detection at 320 kHz. The noise density is 2.5 nV/sqrt(Hz) when there is no probe light on the photodiode which is dominated by the input electrical noise of the LIA. With balanced detection, when the probe power on the photodetector is less than 6 mW, the low noise frequency band is flat <500 kHz. To minimize the input noise of LIA, the input signal must be filtered out at the frequency of the pulse repetition rate. Furthermore, the probe SBG gain at the frequency of the amplitude modulation of the pump needs to have low transmission loss. The pump envelope modulation frequency and the repetition rate of the pulse are selected to 320 kHz and 1.1 MHz so that a low pass filter (LPF-B0R35+, MiniCircuits) meets the above conditions.

The LIA is set to 2nd-order filter with a noise-equivalent bandwidth of 200 Hz with a frequency scan range of 2 GHz and a pixel time of 20 ms. This setting is used for all the cells, organoids, *C. elegans* embryo and mouse embryo imaging. For zebrafish larva imaging, the LIA is set to 2nd-order filter with noise-equivalent bandwidth of 100 Hz, a frequency scan range of 3 GHz and a pixel time of 40 ms. For *C. elegans* imaging in the head and gonad regions, the LIA is set to 2nd-order filter with noise-equivalent bandwidth of 300 Hz, a frequency scan range of 3 GHz and a pixel time of 20 ms. While our chosen frequency scan range of 2-3 GHz is slightly less than that of previous work[1], our simulations (Extended Data Fig. 8) show that the spectral precision with 2-3 GHz scanning range is sufficiently high for both for single Lorentzian and double Lorentzian fitting.

In all the Brillouin imaging experiments, the pulsed-pump envelope modulation frequency is selected at 320 kHz. The pulsed probe beam is with 60 ns pulse width, 1.1 MHz repetition rate and 7 mW average power on the sample (corresponding to ~5 mW average power on the photodiode). The signal and reference beams are connected to the two differential inputs of the LIA with 50 Ω input impedance. For 200 Hz noise-equivalent bandwidth, the noise defined by the standard deviation of the LIA output when the pump-probe frequency difference is tuned away from the Brillouin peak, is measured to be 46.7 nV. Therefore, the measured noise density is 3.30 nV/sqrt(Hz). The average probe power on the photodiode is 5 mW and the responsivity of the photodiode is 0.55 A/W. So the shot-noise density of the single signal beam is $\sqrt{2e\eta P} * R = 1.5$ (nV/sqrt(Hz)), where e is the elementary charge, η is the responsivity of the photodiode (0.55 A/W for the photodiode we use), P is the average power on the photodiode, R is the input impedance of the LIA. The total estimated noise density, including the shot noise of the signal and the reference as well as the input electrical noise of the LIA, is calculated to be 3.28 nV/sqrt(Hz) which is very close to the measured noise. Note that the total noise is ~2 times larger than the shot noise of a single signal input.

SUPPLEMENTARY TABLE

	CPU (Matlab) - Average of 7 repetitions	CPU + GPU (Gpufit) - Average of 10 repetitions	Speedup
Single peak: time per pixel/spectrum	25.8 ms	0.016 ms	x1541
Double peak: time per pixel/spectrum	68.1 ms	0.088 ms	x775
Total time to process 1 plane (30401 pixels total)	2860.64s	8.34 s	x343

SI Table 1: Processing times of the spectral dataset underlying Fig. 3c, which is composed of 301x101=30401pixels. The total processing time also includes additional steps, including loading of the data and computing fit statistics, which are currently only done on the CPU.

SUPPLEMENTARY REFERENCES

1. Remer, I., Shaashoua, R., Shemesh, N., Ben-Zvi, A. & Bilenca, A. High-sensitivity and high-specificity biomechanical imaging by stimulated Brillouin scattering microscopy. *Nat. Methods* **17**, 913–916 (2020).
