# Supplementary material for: Pulsed stimulated Brillouin microscopy enables high-sensitivity mechanical imaging of live and fragile biological specimens
Source: Nat Methods. 2023 Oct 26;20(12):1971–9. doi: 10.1038/s41592-023-02054-z (PMC10703689; doi:10.1038/s41592-023-02054-z)
Supplement: Supplementary file 2 — Reporting Summary [file 41592_2023_2054_MOESM2_ESM.pdf]

nature research

Corresponding author(s): Fan Yang, Robert Prevedel

Reporting Summary

Nature Research wishes to improve the reproducibility of the work that we publish. This form provides structure for consistency and transparency in reporting. For further information on Nature Research policies, see Authors & Referees and the Editorial Policy Checklist.

Statistical parameters

When statistical analyses are reported, confirm that the following items are present in the relevant location (e.g. figure legend, table legend, main text, or Methods section).

n/a	Confirmed	
☐	☒	The exact sample size (n) for each experimental group/condition, given as a discrete number and unit of measurement
☐	☒	An indication of whether measurements were taken from distinct samples or whether the same sample was measured repeatedly
☐	☒	The statistical test(s) used AND whether they are one- or two-sided<br>*Only common tests should be described solely by name; describe more complex techniques in the Methods section.*
☒	☐	A description of all covariates tested
☒	☐	A description of any assumptions or corrections, such as tests of normality and adjustment for multiple comparisons
☐	☒	A full description of the statistics including central tendency (e.g. means) or other basic estimates (e.g. regression coefficient) AND variation (e.g. standard deviation) or associated estimates of uncertainty (e.g. confidence intervals)
☐	☒	For null hypothesis testing, the test statistic (e.g. F, t, r) with confidence intervals, effect sizes, degrees of freedom and P value noted<br>*Give P values as exact values whenever suitable.*
☒	☐	For Bayesian analysis, information on the choice of priors and Markov chain Monte Carlo settings
☒	☐	For hierarchical and complex designs, identification of the appropriate level for tests and full reporting of outcomes
☒	☐	Estimates of effect sizes (e.g. Cohen's d, Pearson's r), indicating how they were calculated
☐	☒	Clearly defined error bars<br>*State explicitly what error bars represent (e.g. SD, SE, CI)*

Our web collection on statistics for biologists may be useful.

Software and code

Policy information about availability of computer code

Data collection	Custom written LabView 2018 control software.
Data analysis	Custom written Matlab R2021b scripts, CUDA 11.5 code (see https://github.com/prevedel-lab/sbs-gpu-acceleration), GPUfit v1.2.0; Fiji 1.52i

For manuscripts utilizing custom algorithms or software that are central to the research but not yet described in published literature, software must be made available to editors/reviewers upon request. We strongly encourage code deposition in a community repository (e.g. GitHub). See the Nature Research guidelines for submitting code & software for further information.

Data

Policy information about availability of data

All manuscripts must include a data availability statement. This statement should provide the following information, where applicable:

- Accession codes, unique identifiers, or web links for publicly available datasets
- A list of figures that have associated raw data
- A description of any restrictions on data availability

The raw datasets generated and/or analysed during the current study are available at https://doi.org/10.5281/zenodo.8211867. Source data are provided with this paper.

Field-specific reporting

Please select the best fit for your research. If you are not sure, read the appropriate sections before making your selection.

☒ Life sciences ☐ Behavioural & social sciences ☐ Ecological, evolutionary & environmental sciences

For a reference copy of the document with all sections, see nature.com/authors/policies/ReportingSummary-flat.pdf

Life sciences study design

All studies must disclose on these points even when the disclosure is negative.

Sample size	This work focused on the development of a new imaging technique. The performance of the microscope was validated on imaging double-distilled water, mouse fibroblast cells, primary human brain microvascular endothelial cells, mouse stem cells, young adult C. elegans worms, zebrafish larvae, C. elegans embryos, mouse embryos and mouse mammary gland organoids. In general, the imaging was repeated between 2 to 30 times on each cell, embryo or organism, all of which produced comparable data quality. These numbers are indicated in the main text and below. This sample size was sufficient in our opinion since we were demonstrating a microscope's performance and were not investigating a biological question. For microscope characterization, a large spectral sample size (n=300) was chosen to obtain Normal distribution.
Data exclusions	No data was intentionally excluded from the study. Representative data sets were chosen for the Figures.
Replication	We repeated the in-vivo imaging experiments multiple times on a total of n=30 individual fibroblast cells, n=5 primary human brain microvascular endothelial cells, n=2 mouse stem cells, n=10 C.elegans worms, n=4 zebrafish larvae, n=5 mouse mammary gland organoids, n=9 C.elegans embryos, and n=10 mouse embryos. The results were reproducible, i.e. they yielded image datasets of comparable quality.
Randomization	We did not preselect or pre-screen samples, but chose them randomly in order to demonstrate the capabilities of our new microscopy technique.
Blinding	In principle we were blinded to any group allocation and the outcome of our study, i.e. the demonstration of a new microscopy technique, is independent of the biological sample studied.

Reporting for specific materials, systems and methods

Materials & experimental systems

n/a	Involved in the study
☒	☐ Unique biological materials
☒	☐ Antibodies
☐	☒ Eukaryotic cell lines
☒	☐ Palaeontology
☐	☒ Animals and other organisms
☒	☐ Human research participants

Methods

n/a	Involved in the study
☒	☐ ChIP-seq
☒	☐ Flow cytometry
☒	☐ MRI-based neuroimaging

Eukaryotic cell lines

Policy information about cell lines

Cell line source(s)	Mouse fibroblast cells: NIH/3T3-CRT-1658 from ATCC;<br>Mouse embryonic stem cells: sox1-GFP-mESCs from Austin Smith's lab, University of Exeter;<br>Primary human brain microvascular endothelial cells: HBMECs from Cell systems.
Authentication	These cell lines have been authenticated by the vendors. In particular, we purchased the NIH/3T3 cells from ATCC where they were authenticated by cytochrome 1 oxidase barcoding for species detection and morphology. The cells have also been tested for mycoplasma and ectromelia virus and were confirmed to be negative for both.<br>The HBMECs from Cell systems were validated by immunofluorescence for endothelial markers.
Mycoplasma contamination	The cell lines used in this study are not contaminated by mycoplasma.
Commonly misidentified lines (See ICLAC register)	There is no misidentified cell line used in this study.

Animals and other organisms

Policy information about studies involving animals; ARRIVE guidelines recommended for reporting animal research

Laboratory animals	This work followed the European Communities Council Directive (2010/63/EU) to minimize animal pain and discomfort. All animal care and procedures performed in this study conformed to the EMBL Guidelines for the Use of Animals in Experiments and were reviewed and approved by the Institutional Animal Care and Use Committee (IACUC), under protocol number 2020-01-06RP. (C57BL/6JxC3H/He) F1 mice from eight-weeks of age onwards were used. Embryos were recovered from superovulated female mice mated with male mice. Mice were maintained in individually ventilated plastic cages (Tecniplast) in an air-conditioned (temperature 22 °C ± 2 °C, humidity 50% ± 10%) and light-controlled room (illuminated from 07:00 to 19:00 h). Mice were fed 1318 P autoclavable diet (Altromin, Germany) ad libitum.<br><br>Wild-type zebrafish larvae (3 days after fertilization) were used in the experiments.<br>N2 Bristol strain C. elegans worms (6 hours post L4 stage) were used in the C. elegans worm experiments. The C. elegans embryos (2 hours post-egg-laying) were transferred using platinum wire onto glass slides with M9 solution.
Wild animals	The study did not involve wild animals.
Field-collected samples	The study did not involve field-collected samples.
